# Access to reproductive health services among the female floating population of childbearing age: a cross-sectional study in Changsha, China

**DOI:** 10.1186/s12913-019-4334-4

**Published:** 2019-08-01

**Authors:** Yanhui Zhou, Ting Wang, Jingxia Fu, Mingzhu Chen, Yanting Meng, Yang Luo

**Affiliations:** Xiangya Nursing School, Central South University, No.172, Tongzipo Road, Changsha, Hunan province 410013 People’s Republic of China

**Keywords:** Reproductive health, Floating population, Female, Service, Utilization

## Abstract

**Background:**

The floating population serves an important role in economic and social development. However, little is known about the floating population’s reproductive health (RH) services, especially in low- and middle-income countries. This study aimed to assess the use of reproductive health services in the female floating population in China, which is a country with the largest floating population in the world.

**Methods:**

A cross-sectional survey was conducted for more than 3 months. Six hundred twenty females of childbearing age in a floating population were recruited into the study by using random sampling, with these individuals being recruited from six community centres in Changsha, China. The use of reproductive health services was assessed by utilizing a self-designed questionnaire.

**Results:**

A total of 555 participants returned the completed questionnaires (effective response rate of 89.5%), including 405 married women and 150 unmarried women. The utilization of RH services was poor in individuals who could access RH policies (39.3%), RH education (36.4%), RH counselling (27.4%), gratis contraceptives (36.0%), and free RH examinations (38.9%), and married women utilized these services at higher rates than unmarried women (*P* < 0.01), although 63.3% of the unmarried women had sexual lifestyles. The marital status was significantly associated with receiving RH education, RH counselling, gratis contraceptives, and free RH examinations. Age was significantly associated with the use of RH education and free RH examinations. The average personal monthly income had a significantly beneficial effect on the use of free RH examinations. Obstetrics and gynaecological disease prevention (67.2%) were the greatest needs of the RH services, and the use of the Internet was the best way to obtain these services. Most of the individuals (77.3%) hoped to receive gynaecological health screenings that were provided by obstetrics and gynaecology hospitals.

**Conclusions:**

The female floating population exhibited poor awareness of RH and rarely used RH services, especially in unmarried women. The results suggest that educational interventions for the female floating population, as well as policy and resource developments should meet the demands for RH services, which are urgently needed in China.

**Electronic supplementary material:**

The online version of this article (10.1186/s12913-019-4334-4) contains supplementary material, which is available to authorized users.

## Background

Reproductive health (RH) is a term used to describe a state of complete physical, mental, and social well-being (and not merely the absence of disease) in all matters related to the reproductive system and its function and processes. RH is associated with human reproduction and development, and it is also related to several major diseases and social problems. The achievement of universal access to RH services for all individuals has been emphasized and accepted worldwide [1]. The global RH strategy was again announced in 2004 by the World Health Organization (WHO), which recommended the monitoring of RH services at the national level [1, 2]. Hence, the enjoyment of RH services is a common right and is important to all human beings.

The floating population (FP), which is a term referring to a main statistical indicator, refers to a population of residence-registration inconsistent individuals, excluding intra-city individuals. Additionally, a population of residence-registration inconsistent individuals refers to those individuals who have been residing in places other than registered streets or towns and who have been away from their registration areas for over half a year [3]. However, FP is different from “migrants”, who frequently change their place of residence and who are mainly referred to as “internal migrants” in a country during this study. According to the 2015 World Migration Report, with a massive population moving to cities, the urban population is expected to grow to approximately 6.4 billion by 2050, which results in both challenges and opportunities, especially in low- and middle-income countries [4]. Although both international and internal migrants make significant and essential contributions to the economic, social and cultural development of the cities in which they live, they are vulnerable to ill-health, such as RH diseases [5–7]. Previous studies have indicated that migrant women use less RH services than non-migrant women, in terms of prenatal care, hospital deliveries, postpartum visits, and system administration [8–11].

China, the largest developing country in the world, has witnessed a significant increase in FP individuals in recent decades. Based on the “Report on the development of China FP in 2016”, the total number of FP individuals is expected to gradually reach close to 300 million before 2020, and most of these individuals are concentrated in the Yangtze River Delta, the Pearl River Delta, Beijing-Tianjin-Hebei, and three other urban agglomerations [12]. However, large numbers of FP individuals who are surging into urban areas are substantially aggravating the burden of economic, social, and, in particular, health issues, thus further leading to the existence of the gaps between the FP and the local population in terms of access to RH services. A quantitative comparative study in Guangzhou city reported that 9.7–35.8% of the FP had no knowledge of at least one RH skill (RH skills for pregnancy tests, contraceptives, the cleaning of genital tracts, maternal nutrition during pregnancy, miscarriage prevention, early education for the foetus, and safe sex), and the frequency of using family planning services (FPS) among the FP was low [13]. One survey that was performed in three manufacturing factories in Guangzhou and Shenzhen cities demonstrated that the rate of premarital sexual intercourse was 17% and that the pregnancy rate was 26.4% for unmarried women of the FP [14]. Another study of 4037 females in the FP, with a history of pregnancy, from 5 cities in China observed that 24.7% (996/4,037) did not receive the benefit of prenatal health care services [15]. In addition, studies have reported that the needs for RH among unmarried women remained poorly understood in most developing countries [16–18]. Such findings reveal that females in the FP are lacking in RH knowledge and skills, that the provision of RH services is not ideal, and that the utilization rate of RH services remains low in China, especially for unmarried female FP.

In 1999, the Millennium Development Goals reaffirmed that global access to RH services should be a priority. As a member of the United Nations, China has issued a series of policies to promote the basic public services of RH, which enables the FP to receive the same propaganda, education, services, and management that are included in the RH services, including policy propaganda, health education, health consulting, contraceptive and birth control services, prenatal and postnatal care services, breast examinations, and cervical cancer screenings. Moreover, most of the FP individuals were likely to suffer from reproductive problems, on account of their characteristics of being of childbearing age, having lower educational levels, having poor health awareness, and having poor living and working environments [19]. Consequently, females in the FP have encountered severe challenges in the RH. Changsha, a provincial capital of Hunan province and an important provincial capital city in central China, is adjacent to the Yangtze River Delta and the Pearl River Delta. This city, which is a part of the first batch of 40 pilot cities offering basic public services of family planning (in which all FP individuals can receive RH services that are not limited by household registration), has attracted increasing numbers of FP individuals because of the rapid economic development in the last decade. However, the use of reproductive health services among the FP was unknown after the institution of the new policy. Therefore, the study of the accessibility and availability of RH services among the FP in Changsha is of great significance. Emerging data have demonstrated the existence of health disparities between rural and urban populations, between migrants and non-migrants, and between married and unmarried women in the use of RH services [13, 15, 20]. However, domestic studies in China have shown that the relevant studies mainly focused on the coastal areas and the Pearl River Delta region, whereas married females in the FP and unmarried females in the FP who are located in the central areas of China are rare examples. Thus, further studies are needed to add research evidence concerning access to RH services of the FP in the central areas of China, in order to meet the needs of RH services for the FP.

The objective of this study was to assess the use of RH services among females in the FP who were of childbearing age in Changsha city, with such services including RH-related knowledge, skills, utilization, and needs. The results of this study may motivate policymakers to adopt suitable approaches to ameliorating the use of RH services of the FP.

## Methods

### Design

A cross-sectional survey was performed among females in the FP who were of childbearing age by using a self-administered questionnaire during a 3-month period from July to September of 2015.

### Sample and setting

The survey was conducted in Changsha, Hunan province, China. Changsha, the capital and the city with the highest proportion of floating population individuals, is located in Hunan province and has a population density of 647 people/km^2^ [21].

It consists of 6 districts, 1 county, and 2 county-level cities, according to the geographical location. Six communities were selected as representative samples for this study because they have a similar floating population structure, thus enabling data collection in the time frame of the study.

The target population in this survey consisted of females in the FP who were living in the community. When considering the accessibility of the participants, a three-step sampling approach was used for the purpose of this study in order to minimize selection bias and to ensure generalizability. First, the community with the FP was stratified into six districts. Second, one community from every district was randomly selected by using a random number generator. Third, all of the females in the FP who were living in the selected community were invited to participate in the present study by the community’s health services staff and via recruiting posters. The following inclusion criteria were applied: 1) female in the FP; 2) females who had registered residences (hukou) that were not in Changsha; 3) females who were aged from 18 to 50 years old; 4) females who were residing in Changsha city for more than 6 months; and 5) females who provided oral informed consent. In China, according to the geographical location of the official residence of the householder and the occupations of the parents, a household registration system in the 1950s divided the country residents into two groups: urban households and rural households (hukou) [13]. The lower age limit was applied to avoid the need to obtain parental consent, whereas the upper age limit was applied because it represents the upper end of natural fertility [13, 20]. The duration time of more than 6 months originated from the definition of the FP and was also developed to better understand the utilization of RH services. The exclusion criteria were as follows: 1) females who were not able to read or answer the questionnaires (e.g., who had difficulties with the language or who had dementia) and 2) females who were studying in Changsha city as students.

The questionnaire survey was conducted after a lecture concerning legal knowledge (the Labour law) that was given at the same time in the three communities, in order to avoid contamination of the participants. The lecture was presented to the participants as a gift. Before being asked to complete a 15-min self-questionnaire concerning RH services, one of the trained researchers (consisting of three graduate students) briefly discussed the purpose of the study in layman’s terms. Participation was voluntary and anonymous, and the participants were allowed to submit a blank questionnaire if they desired. The questionnaires were delivered in envelopes and were immediately collected after they were completed.

### Measurements

The preliminary items were compiled after the review of previous studies [13, 15, 16, 22] and after consultations with 7 relevant experts (three nurses, two women’s health experts, and two public health experts), and the items were then piloted by 30 women in the community. The final 28-item questionnaire was divided into 3 parts: demographic information (7 items), the use of RH services (17 items), and the needs for RH services (4 items). The questionnaire was developed for this study and didn’t have previously been published elsewhere. (see Additional file 1 for more details).

### Demographic data

A demographic questionnaire was developed to collect participant information for items related to age, marital status, education, professional situation, household type (hukou), the duration of staying in the current residence, and monthly income.

### Use of RH services and needs for RH services

#### Use of RH services

1) Do/did you know the RH-related policies? (“yes” or “no”); 2) Do/did you have access to get RH-related health education? (“yes” or “no”); 3) Do/did you have access to get RH counselling? (“yes” or “no”); 4) Do/did you have access to get gratis contraceptives? (“yes” or “no”); and 5) Do/did you have access to obtain free RH-related examinations? (“yes” or “no”). If an individual chose “yes”, then she would continue to answer the following questions: whether or not she had gotten free pregnancy screenings, premarital health checks, breast examinations, B-ultrasonic examinations of the ovaries and uterus, leucorrhoea routine examinations, and cervical smear examinations, and where she had gotten the examinations (the family planning services station, the community hospital, the maternal and child health care centre in the district, municipal level and above hospitals).

#### Needs for RH services

What type of RH-related knowledge and how she most like to get this knowledge, as well as what type of RH-related service and she most like to get the service.

### Data analysis

The data were checked for errors before a double-entry computer input. Descriptive statistics were used to examine the socio-demographic factors and the statutes of the RH services of the samples, including means and standard deviations (SDs) that are represented as percentages. The *t* test was used for the measurement of the variables, and the chi-square statistic was used for the measurement of the categorical variables, in order to assess the statistical significances among the groups. The differences between the married and unmarried women for the variables that represented the count data were analysed by using the *X*^*2*^ test. The binary logistic regression analysis with the selection method was used to identify the dependence of the use of RH services on the independent variables. These variables included age, marital status, and other variables. Logistic regression analyses were performed to examine the correlation factors of the utilization of RH services. Enter methods were used to select the variables that were correlated with the five aspects of using RH services. The inclusion *P*-value was 0.05, and the removal value was 0.10. A *P*-value < 0.05 was considered to be statistically significant. The software package SPSS Statistics Version 19.0 (IBM Corp., Armonk, New York, USA) was used for all of the analyses.

### Ethical consideration

Ethical review and approval were provided by the Hunan Research Ethics Committee of Xiangya School of Nursing, Central South University (Project Number 2015089). Informed consent was obtained from all of the individual participants who were included in the study. A cover letter and the questionnaire in Chinese were distributed to explain the aims and processes of the study. Participation was voluntary and confidential. The returning of the questionnaire was a voluntary way of participating in the study; thus, no consent form was required. To maintain anonymity, the participants were asked to leave the completed questionnaire in a box that was left at the community service centre. Moreover, a law lecture (concerning the Labour law) was conducted in every community to improve the FP’s awareness of the law, with this lecture being provided as a gift to thank the individuals for their participation. All of the collected data were anonymously and confidentially treated.

## Results

### Participants’ demographic characteristics

Among the 620 women who were present, 572 questionnaires were returned (response rate: 92.2%). Blank questionnaires (*n* = 17) were excluded from the data analysis, thus resulting in a final study population of 555 (effective response rate = 89.5%). Most of the respondents were married women (73.0%) who were from villages (65.6%) and who had lived in Changsha for more than 36 months (68.3%). The average age of the participants was 31.45 years (SD = 8.0), and approximately 53.9% of them were between 21 and 32 years old. Of the individuals, the average personal monthly income (RMB) was between 1001 and 3000 (63.8%). The demographic characteristics of the participants are summarized in Table 1.

**Table 1:** Demographic characteristics of female FP (*N* = 555)

Characteristics	Total *N* = 555 (%)	Unmarried *N* = 150 (%)	Married *N* = 405 (%)	*P*-value
Age (years)				0.000^*^
≤ 20	62 (11.2)	34 (22.7)	28 (6.9)	
21–26	186 (33.5)	86 (57.3)	100 (24.7)	
27–32	113 (20.4)	27 (18.0)	86 (21.2)	
33–38	78 (14.4)	3 (2.0)	75 (18.5)	
39–44	70 (12.6)	0 (0.0)	70 (17.3)	
≥ 45	46 (8.3)	0 (0.0)	46 (11.4)	
Education level				0.034^*^
Illiterate/No formal education	3 (0.50)	1 (0.7)	2 (0.5)	
Elementary school	27 (4.9)	1 (0.7)	26 (6.4)	
Junior middle school	165 (29.7)	40 (26.7)	125 (30.9)	
Senior middle school/Polytechnic school	134 (24.1)	37 (24.7)	97 (24.0)	
University or above	226 (40.7)	71 (47.3)	155 (38.3)	
Occupation				0.000^*^
Worker in the factory	151 (27.2)	47 (31.3)	104 (25.7)	
Service industries	140 (25.6)	39 (26.0)	103 (25.4)	
Company employee	94 (16.9)	38 (25.3)	56 (13.8)	
Self-employment venture	94 (14.2)	10 (6.7)	69 (17.0)	
Teacher/Medical worker	27 (4.9)	8 (5.3)	19 (4.7)	
Awaiting job Assignment/laid-off workers	62 (11.2)	8 (5.3)	54 (13.3)	
Monthly income (RMB)				0.000^*^
< 1000	8 (1.4)	3 (2.0)	5 (1.2)	
1000–1999	185 (33.3)	15 (10.0)	170 (42.0)	
2000–2999	169 (30.5)	69 (46.0)	100 (24.7)	
3000–3999	106 (19.1)	45 (30.0)	61 (15.1)	
≥ 4000	87 (15.7)	18 (12.0)	69 (17.0)	
Household type (hukou)				1.000
City or town	191 (34.4)	52 (34.7)	139 (34.3)	
Village	364 (65.6)	98 (65.3)	266 (65.7)	
Duration of residence (months)				0.000^*^
6–12	65 (6.0)	28 (18.7)	3 (0.8)	
13–24	81 (15.5)	41 (27.3)	40 (10.8)	
25–36	53 (10.2)	19 (12.7)	34 (9.2)	
> 36	356 (68.3)	62 (41.3)	294 (79.2)	
Having a sexual lifestyle	–	95 (63.3)	–	–

^*^P ≤ 0.05 indicates statistical significance

### Use of RH services

The utilization of RH services mainly included five aspects in this study: 1) awareness of RH policies, 2) access to receive RH education, 3) access to acquire RH counselling, 4) access to gain gratis contraceptives, and 5) access to obtain free RH examinations. Table 2 indicates that the number of married women receiving RH services was more than that of unmarried women. Statistical significance existed between them in all aspects. However, the total proportion of females receiving RH services was low (less than 40%). The highest rate was the awareness of RH policies (39.3%), and the lowest rate was the access in acquiring RH counselling (27.4%).

**Table 2:** The use of RH services among female FP

RH services	Total *N* = 555 (%)	Unmarried *N* = 150 (%)	Married *N* = 405(%)	*P*-value
RH-related policies				0.001^*^
Yes	218 (39.3)	42 (28.0)	176 (43.5)	
No	337 (60.7)	108 (72.0)	229 (56.5)	
RH education				0.000^*^
Yes	202 (36.4)	33 (22.0)	169 (41.7)	
No	353 (63.6)	117 (78.0)	236 (58.3)	
RH counselling				0.000^*^
Yes	152 (27.4)	10 (6.7)	142 (35.1)	
No	403 (72.6)	140 (93.3)	263 (64.9)	
Gratis contraceptives	*n* = 500	*n* = 95		0.000^*^
Yes	180 (36.0)	8 (8.4)	172 (42.5)	
No	320 (64.0)	87 (91.6)	233 (57.5)	
RH examinations				0.000^*^
Yes	216 (38.9)	21 (14.0)	195 (48.1)	
-Pregnancy screening		–	172 (88.2)	
-Premarital health check		–	80 (41.0)	
-B-ultrasonic examination of the ovaries and uterus		20 (95.2)	158 (81.0)	
-Leucorrhoea routine examination		15 (71.4)	154 (79.0)	
-Breast examination		18 (85.7)	129 (66.2)	
-Cervical smear examination		–	116 (59.5)	
No	339 (61.1)	129 (86.0)	210 (51.9)	

^*^P ≤ 0.05 indicates statistical significance

### Predictors for the utilization of RH services

Table 3 presents the results of the logistic regression analysis on the relationship between the use of RH services (including the five aspects) and the demographic characteristics of the female FP. The marital status was significantly associated with the use of obtaining RH education, RH counselling, gratis contraceptives, and free RH examinations. Age was significantly associated with the use of RH education and free RH examinations. The average personal monthly income had a significantly beneficial effect on only the free RH examinations.

**Table 3:** Social determinants of RH services among female FP

Social determinants of RH services	Coefficient	OR	95% CI		*P*-value
			Lower	Upper	
RH-related policies					
Resident time	0.404	1.498	1.305	1.719	0.000^*^
RH education					
Age	0.035	1.035	1.002	1.070	0.038^*^
Marital status	1.223	3.397	1.708	6.757	0.000^*^
Resident time	0.670	1.954	1.200	3.183	0.007^*^
RH counselling					
Marital status	1.170	3.222	1.056	9.827	0.040^*^
Gratis contraceptives					
Marital status	1.405	4.077	1.929	8.615	0.000^*^
RH examination					
Age	0.601	1.824	1.490	2.234	0.000^*^
Marital status	1.471	4.354	2.061	9.198	0.000^*^
Resident time	0.786	2.195	1.343	3.588	0.002^*^
Monthly income	−0.586	0.556	0.428	0.723	0.000^*^

*CI* Confidence interval, *OR* odds ratio

^*^P ≤ 0.05 indicates statistical significance

### Needs for RH services

The findings revealed that the main contents of the needs for RH knowledge and skills were obstetrics and gynaecological disease prevention (67.2%), policies and laws related to the FP (37.2%), prepotency (30.3%), pregnancy and postpartum care (24.9%), contraceptives and birth control (18.6%), and sexual knowledge (9.2%). However, 2.8% of them claimed it to be unnecessary. Afterwards, the primary ways of acquiring RH knowledge were Internet searches (45.2%), propaganda books or booklets (36.8%), RH lectures (36.4%), television (32.4%), face-to-face counselling (31.7%), door-to-door education (24.7%), and telephone consulting (20.0%).

**Fig. 1:**
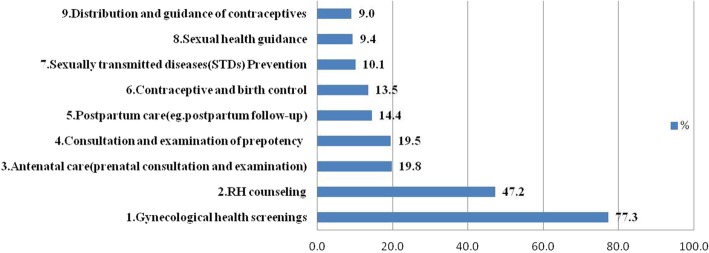
The needs for basic RH services. The percentage of participants who most like to receive each kind of RH-related service

Figure 1 shows the needs for basic RH services, with more than three-quarters of the respondents hoping to obtain gynaecological health screenings provided by the obstetrics and gynaecology hospital.

## Discussion

The present study indicated that the unmarried and married females in the FP were different in terms of the social and demographic characteristics, including educational level, occupation, monthly income, and residence time. Most of the females in the FP were sexually active young women with higher education levels, middle income, occupations as labourers and in service work, and rural household registrations, which confirmed that the FP had poor life and job conditions; however, the education level was higher in this study than in previous studies [23–25]. In this study, more than half of the unmarried women had sexual lifestyles. This finding supports the results of previous studies that the rate of premarital sex among unmarried females in the FP was high [13, 15, 16, 22], and this rate was higher than the rate in the study of unmarried rural-urban females in the FP in Shanghai, China [26]. Such behaviour was likely to increase the prevalence of accidental pregnancies and induced abortions among unmarried women and could also increase the risks of reproductive tract infections (RTIs) and sexually transmitted diseases (STDs). In fact, premarital sexual intercourse is a behaviour resulting from the interaction of family, social, personal, and environmental factors [14]. Previous studies have revealed that unmarried females in the FP were characterized by having poor recognition of safe sex, as well as a lack of essential economic and medical securities [18, 27, 28]. Our research showed that several participants were awaiting job assignments or had lower income (with some individuals even having a lack of income); thus, they were more vulnerable to poverty. Furthermore, the different social environment promoted different attitudes concerning sex-related behaviours [29, 30]. Some unmarried women felt embarrassed to discuss sexual matters, which is caused by traditional awareness, and did not seek help for RH problems. The ignorance concerning RH also highlights the problems of reproductive safety. Therefore, the unmarried female FP should receive urgent attention.

This study revealed that the use of RH services still remains as a negative factor among the female FP. Compared with the married women, unmarried females in the FP had much lower accessibility of RH education, counselling, gratis contraceptives, and free RH examinations. The findings support the results of a previous study that the RH services of the unmarried FP remain inadequate compared with the married FP in three major cities (Wuhan, Guangzhou, and Shenzhen) in China [11]. In this study, the FP with a young age and low income had a decreased chance of receiving free RH examinations, and age also affected the FP in accessing RH education, which differs from other studies. A number of factors appear to have contributed to these results. First, the rate of sex education persistently lagged. Our study demonstrated that only 27.4 and 36.4% of individuals accepted RH counselling and RH education, respectively, although 40.7% of individuals had education levels at the university level or above, which indicated an inadequacy in sex education. Second, the mandatory long-term contraception policy had dominated. It is well known that the one-child policy, which is a strict family planning policy, had dominated the Chinese family planning programme for more than 30 years [31]. The majority of married couples were allowed to have only one or two children. Additionally, diverse contraceptive methods have been provided in FPS institutions at all levels and have been applied to married people of childbearing age. However, unmarried women were not involved; thus, they denied themselves RH, especially in terms of gratis contraceptives and RH examinations. Third, embarrassment emerged as a personal barrier on the side of the participants [32]. Most people are aware of the necessity of sex education, but they feel embarrassed or have a fear of stigma in discussing the issue of sex and RH, especially in unmarried women [33, 34]. With regard to RH examinations, vaginal and cervical cancer examinations were considered to be personally private, inconvenient, and painful. Therefore, the rate of RH examinations was low, and unmarried women received a lower number of RH examinations than married women in this study. Finally, poor awareness was the fourth important barrier to RH services. Some females in the FP believed that an RH examination was not important and even overestimated their RH level. However, the epidemics of RTIs and STIs remained high and were linked to the increased mobility of the population [6, 35, 36]. Additionally, a routine leucorrhoea examination, which is a simple method of evaluating the reproductive health of women, refers to the use of sterile cotton swabs that are dipped in leucorrhoea for laboratory examinations with the use of a vaginal endoscope. Moreover, a cervical smear test is an essential item for a gynaecological examination and is the most convenient and effective method for the diagnosis of cervical cancer. If these RH examinations were not conducted, then RH-related diseases would be hard to diagnose. Therefore, the poor utilization of RH services reveals that health education provided little information on sexual issues to the female FP. An effective intervention should be formulated, including correct and comprehensive information for RH, as well as a set of feasible schemes and accurate assessment methods for RH education.

According to the findings, those females in the FP with longer durations of stays in the current residence, as well as females with older ages who were married and had a higher income per month were more likely to utilize the RH services. The differences in the socio-demographic characteristics may partially explain the five aspects of RH service utilization, which is similar to previous studies in other countries [7, 37, 38]. This may be caused by the health awareness and economic positions of other countries. One report indicated that females in the FP with a higher income (above 2000 RMB) had higher uses of RH services compared with those females with a lower income. Additionally, the higher educational levels of the participants indicated a better understanding of RH knowledge [13]. The RH education levels among the lower income and lower education populations should be urgently promoted [39, 40]. Furthermore, a qualitative study observed that the behaviours of floating women were influenced by the precepts of their origin societies [29]. Therefore, effective RH service mechanisms should be adapted to the social-demographic characteristics. In this regard, even though the Chinese government has proposed a plan to promote equal access to basic public services for the FP and has conducted pilot work in many provinces, it is necessary to further improve the accessibility and availability of public RH services to ameliorate the utilization of these services.

In addition, this study suggests that the providers of RH services should consider the needs of the female FP, such as the specific contents of RH knowledge and skills, and the ways of acquiring RH knowledge. At present, a mobile health (m-Health) programme has been widely recognized as being an effective method for improving health services. It is evident in the literature that an e-health intervention for the internal floating population in Vietnam can effectively increase the knowledge of the female population regarding sexual lifestyle and RH factors, as well as foster improved practices that are related to sexual lifestyle and RH factors [41]. Our findings indicated that the majority of participants tended to use “online” approaches for receiving RH-related information. The likely reason for this result was due to the ease in obtaining Internet access without limitations of time and space. Moreover, other ways of obtaining RH-related education should be given more attention to satisfy the different groups, including propaganda books or booklets, lectures, television, face-to-face counselling, door-to-door education, and telephone consulting. Hence, the governmental departments should take actions to provide appropriate, specific, friendly, and accessible services for floating women.

Recently, the global RH strategy and the policy of the equalization of basic public services in China advocate that the provisions of services for the FP should be similar to those provided for the local residents. However, a well-developed RH service programme that targets the female FP is deficient, especially with regard to unmarried women. The government and providers need to take action to embed the awareness of RH public services into the FP. Further research should be conducted to identify the appropriate strategies for improving the use of RH services and to translate them into practice, in order to improve the RH for the FP.

The findings from this study should be interpreted in the context of its limitations. First, the questionnaire was compiled by reviewing previous studies and consulting experts, and the reliability and validity of the study were only tested by consulting experts. Second, the predominant outcomes were self-reported by the female FP and were not objectively measured or confirmed by medical reports. Nevertheless, anonymity and confidentiality were emphasized, and the participants were encouraged to truthfully answer all questions. Third, all of the participants were invited by the community health services staff and via recruiting posters in the community, and they were asked to complete the questionnaire after the law lecture. However, a selection bias still exists. More studies should be conducted to further highlight the opportunities for improving RH care services.

## Conclusion

In general, the awareness regarding RH and the use of RH services among the females in the FP in Changsha cities remains insufficient, especially with regard to the unmarried female FP. This study highlighted the need for tailored interventions. The findings provide evidence that can assist decision makers in bridging the coverage gaps. Online education may be one of the best and effective methods for the FP in enhancing RH knowledge and skills, as well as in improving the use of RH services. Moreover, further research should focus more on the RH service providers than on the users. Further improvement of the RH knowledge and skills of the female FP would certainly complete the truly universal RH coverage in China.

## Additional file

Additional file 1: The questionnaire of access to reproductive health services. (DOC 62 kb)

## Data Availability

The datasets generated and/or analysed during the current study are not publicly available due to this study is one small portion of our project but are available from the corresponding author on reasonable request.

## Abbreviations

FP: Floating population
FPS: Family planning services
RH: Reproductive health
RTI: Reproductive tract infection
STD: Sexually transmitted disease
