# Supplementary material for: Access to reproductive health services among the female floating population of childbearing age: a cross-sectional study in Changsha, China
Source: BMC Health Serv Res. 2019 Aug 1;19:540. doi: 10.1186/s12913-019-4334-4 (PMC6676621; doi:10.1186/s12913-019-4334-4)
Supplement: Supplementary file 1 — The questionnaire of access to reproductive health services. (DOC 62 kb) [file 12913_2019_4334_MOESM1_ESM.doc]

**Additional file1：The questionnaire of access to reproductive health services**

Dear Female friend:

Thank you very much for participating in this questionnaire survey about reproductive health (RH) services. The study is carried out by the Reproductive Health Studies team on the Xiangya Nursing School of Central South University.

We want to know about the access to reproductive health services in Changsha city by scientific inquiry. There is no right or wrong answer. And then, you don’t worry about your information being leaked because the questionnaire is anonymous and used for analysis. We hope that you can spare your precious time and fill in this questionnaire according to your actual situation. If you complete the questionnaire, please leave it in a box named questionnaire bins at the community service centre. If you do not wish to answer any questions, or wish to exit the field at any time, please leave it in the box too.

We would like to express our heartfelt thanks to you for your cooperation and support. Best wishes!

The Reproductive Health Studies team

Xiangya Nursing School of Central South University

**Part 1 Personal Demographic characteristics**

***Instructions:*** *below are some demographic characteristics about you. Please fill in the blank space or tick the appropriate box.*

1. Your age (years):

2. Your marital status □ Married

□ Unmarried: have you ever had sex?? □Yes □No

3. Your educational level:

□ Illiterate/No formal education

□ Elementary school

□ Junior middle school

□ Senior middle school / Polytechnic school

□University or above

4. Your Occupation:

□ Worker in the factory

□ Service industries

□ Company employee

□ Self-employment venture

□ Teacher/Medical worker

□ Awaiting job

□ Assignment/laid-off workers

5. Your monthly income (RMB):

□ < 1000

□ 1000–1999

□ 2000–2999

□ 3000–3999

□ ≥ 4000

6. Your household registration type (hukou):

□ City or town

□ Village

7. How long have you lived or worked in Changsha city? (Duration of residence)

□ 6–12 months

□ 13–24 months

□ 25–36 months

□ > 36 months

**Part 2** **The use of RH service**s

***Instructions:*** *below are some* *statements concerning reproductive health services. Please indicate whether you have accepted the relevant services by ticking the appropriate box.*

8. Do you know the RH-related policies? □ Yes □ No

9. Do you have access to the RH-related health education? □ Yes □ No

10. Do you have access to the RH counselling? □ Yes □ No

11. Do you have access to get free contraceptives? □ Yes □ No

12. Do you have access to free RH-related examinations? □ Yes □ No

***If you choose “yes” for question 12, please continue to answer the following questions:***

| Items | Yes | No |
| --- | --- | --- |
| 13.Have you ever had pregnancy screenings? |  |  |
| 14. Have you ever had premarital health checks? |  |  |
| 15. Have you ever had breast examinations? |  |  |
| 16. Have you ever had B-ultrasonic examinations of the ovaries and uterus? |  |  |
| 17. Have you ever had leucorrhoea routine examinations? |  |  |
| 18. Have you ever had cervical smear examinations? |  |  |

***Where do you get the following RH-related related examinations?***

| Items | Family planning service station | Community hospital | Maternal and child health care centre in the district | Municipal level and above hospitals |
| --- | --- | --- | --- | --- |
| 19. Pregnancy screenings |  |  |  |  |
| 20. Premarital health checks |  |  |  |  |
| 21. Breast examinations |  |  |  |  |
| 22 B-ultrasonic examinations of the ovaries and uterus |  |  |  |  |
| 23. Leucorrhoea  routine examinations |  |  |  |  |
| 24. Cervical smear examinations |  |  |  |  |

**Part 3 The needs for RH services**

***Instructions:*** *below are some statements concerning the needs for reproductive health services. Please indicate your opinion by ticking the appropriate box.*

25.What type of RH-related knowledge or skill would you like to know? ([multiple](javascript:;)-[choice](javascript:;), a maximum of three choices)

□ Family planning policy

□ Policies and laws related to the floating population

□ Sexual knowledge

□ Knowledge of Contraceptives and birth control

□ Knowledge of prepotency

□ Obstetrics and gynaecological disease prevention

□ Pregnancy and postpartum care

□ Unnecessary

26.Which channel would you like to obtain RH-related knowledge? ([multiple](javascript:;)-[choice](javascript:;), a maximum of three choices )

□Television

□ RH lectures

□ Propaganda books or [booklet](javascript:;)s

□ Door-to-door education

□ Face-to-face counselling

□ Telephone consulting

□ Internet searches

□ Others (please specify)

27.What kind of RH-related service would you most like to receive in your current residence? ([multiple](javascript:;)-[choice](javascript:;), a maximum of three choices )

□ RH counselling

□ Gynecological health screenings

□ Contraceptive and birth control

□ Distribution and guidance of contraceptives

□ Sexually transmitted diseases (STDs) Prevention

□ Consultation and examination of prepotency

□ Antenatal care (prenatal consultation and examination)

□ Postpartum care (eg. postpartum follow-up)

□ Sexual health guidance

□ Others (please specify)

28. Which institution would you most like to receive RH services from in your current residence? ([multiple](javascript:;)-[choice](javascript:;), a maximum of three choices )

□ Family planning services department □ Community hospital

□ Obstetrics and gynaecology hospital □ [General](javascript:;) [hospital](javascript:;)

□ Community or street hospitals □ Private hospital

□ Others (please specify)
